# The intervention effect of structured martial arts games on behavioral impairments and motor functions in children with autism spectrum disorder

**DOI:** 10.3389/fpsyg.2025.1660040

**Published:** 2025-09-26

**Authors:** Xuexiang Fu, Peng Shi

**Affiliations:** ^1^School of Physical Education and Health, Huaihua University, Huaihua, China; ^2^School of Physical Education, Shandong University of Technology, Zibo, Shandong, China

**Keywords:** autism, physical games, structured teaching, gross motor function, martial arts, rehabilitation, children

## Abstract

**Objective:**

This study aims to explore the intervention effect of an intervention program based on structured martial arts games on behavioral and motor function disorders in children with autism spectrum disorder (ASD), and to provide a novel and effective intervention strategy for ASD children to enhance their rehabilitation outcomes.

**Methods:**

A randomized controlled trial design was adopted. Fifty-six ASD children were selected from a special education school and randomly divided into an experimental group (28 people) and a control group (28 people). The experimental group received a 24-week structured martial arts game intervention, 3 times a week, 60 min each time; the control group received traditional rehabilitation training. The autism treatment evaluation checklist, autism behavior checklist, and gross motor function measure were used to evaluate the relevant indicators of the two groups of children before and after the intervention, and SPSS 26.0 software was used for statistical analysis.

**Results:**

After the intervention, the improvement of the experimental group in language ability, social communication ability, perceptual ability, health behavior and total score was significantly better than that of the control group (*p* < 0.05). The experimental group was significantly better than the control group in sensory ability, social ability, motor ability, language ability, self-care ability and total score (*p* < 0.05). The improvement of the experimental group in lying and rolling, sitting, crawling and kneeling, standing, walking, running and jumping, and total score was significantly higher than that of the control group (*p* < 0.05).

**Conclusion:**

The structured martial arts game intervention can effectively improve the behavioral and motor disorders of ASD children. This intervention method is both interesting and structured, and can be used as a new approach for the rehabilitation of ASD children. It is recommended to incorporate it into the regular curricula of special education schools and rehabilitation institutions.

## 1 Introduction

Autism spectrum disorder (ASD) is a neurodevelopmental disorder characterized mainly by social impairments, delayed language development, repetitive and stereotyped behaviors, and restricted interests (Mukherjee, 2017; Oberman and Kaufmann, 2020). These symptoms usually emerge in early childhood and have a significant negative impact on an individual’s daily life, learning, and social functioning (Tobin et al., 2014). Salari et al. (2022) evaluated 74 studies involving 30,212,757 participants and found that the global prevalence rate of ASD reached 0.6% (95% *CI* = 0.4 ~ 1.0%). In addition, a subgroup analysis revealed that the prevalence rate of ASD in the United States was 1.0%, and in Asian countries, it was 0.4%. This suggests that ASD imposes a heavy health burden on countries around the world, and it is necessary to implement appropriate programs and intervention measures to alleviate this burden on individuals with ASD.

Behavioral impairments and motor dysfunction are significant characteristics of children with ASD. Children with ASD exhibit multidimensional behavioral impairments, including communication disorders, abnormal social behavior, delayed language development, and repetitive stereotyped behaviors (Bhat, 2023; Bhat et al., 2022), which severely affect their daily learning and life. In addition, children with ASD have gross motor dysfunction (Liu et al., 2014), especially with generally weak balance and coordination abilities (Fournier et al., 2010; Stins and Emck, 2018). They have unstable gaits when walking, uncoordinated running postures, and obvious difficulties in activities such as jumping and climbing. These motor impairments not only affect the self-care abilities of children with ASD in daily life but also further restrict their social participation (Rosenberg et al., 2017). Due to their lagging motor skills, they are often at a disadvantage in sports activities and group games, which can lead to social isolation from peers and consequently exacerbate their social withdrawal behaviors, forming a vicious cycle that severely affects their mental health and social adaptability (Rosenberg et al., 2017). Therefore, how to improve the behavioral impairments and motor dysfunction of children with ASD is a topic of general concern among researchers.

With the deepening of research, physical exercise, as an auxiliary intervention measure, has gradually attracted attention. A growing body of studies (Lang et al., 2010; Sowa and Meulenbroek, 2012; Toscano et al., 2022) indicates that physical exercise can effectively reduce behavioral and motor coordination impairments in children with ASD. Sowa and Meulenbroek (2012) included 13 original studies to evaluate the promoting benefits of structured physical exercise for ASD patients, and found that physical exercise has a positive promoting effect on the improvement of social skills and motor performance of ASD patients. Lang et al. (2010) conducted a systematic review based on 18 studies and found that exercise intervention can effectively reduce the repetitive stereotyped behaviors, aggressive behaviors and off-task behaviors of ASD patients, and can effectively increase the learning response and appropriate motor behaviors (such as playing catch games) of ASD patients. For another example, in relevant empirical studies, Toscano et al. (2022) found that a 48-week exercise intervention significantly reduced the social interaction, attention disorders, repetitive stereotyped behaviors and motor function disorders of ASD patients. In conclusion, physical exercise, as a non-pharmacological intervention measure, has important value in the rehabilitation of children with ASD, providing new ways and methods to improve their behavioral and motor coordination impairments.

Sports games are a form of activity that integrates exercise, play, and educational elements, and they play a unique role in the rehabilitation training of children with ASD. Through sports games, children with ASD can improve their behavioral impairments and motor function disorders in a relaxed and pleasant atmosphere (Qi et al., 2024). Martial arts, as a traditional Chinese sports event, possess unique cultural connotations and fitness values. Currently, existing studies (Bell et al., 2016; Phung and Goldberg, 2021) have utilized martial arts as an intervention method and confirmed that martial arts can enhance the social and behavioral functions of children with ASD. However, due to the fixed nature of current martial arts routine practices, children may find it boring during practice, which reduces their interest in practicing and thus restricts the intervention effect. By combining martial arts with games to form structured martial arts games, this limitation can be overcome. Structured martial arts games offer an environment with predictability for children with ASD through clear rules, an orderly process, and rich movement designs, which help them, concentrate and participate in the activities. Moreover, the interactive elements in martial arts games, such as partner drills and teamwork, can promote communication and cooperation between children with ASD and others, improving their social interaction skills.

Based on this, this study aims to propose an intervention program based on structured martial arts games and explore its intervention effect on behavioral and motor coordination impairments in children with ASD. It is expected to offer a new and effective intervention method for the rehabilitation training of children with ASD, enrich the research content in the field of ASD rehabilitation, and contribute to improving the quality of life and social integration ability of children with ASD.

## 2 Methods

### 2.1 Participants

This study used G • Power 3.1.9.7 software to calculate the sample size. A systematic review and meta-analysis by Wu et al. (2024) found that exercise had a positive effect on improving symptoms related to ASD, with effect sizes ranging from 0.76 to 1.72. Based on this, this study extracted these effect sizes, set *α* at 0.05 and 1 − β at 0.80, and calculated that 12–46 participants were needed. Considering potential dropouts during the intervention, this study calculated that 56 participants were required based on a 20% dropout rate. The participants in this study were ASD children from a special education school in Chengdu, Sichuan Province. To ensure the scientific validity of the study, participant selection followed strict criteria and procedures.

Firstly, professional doctors diagnosed the children according to the diagnostic criteria in the Fifth Edition of the Diagnostic and Statistical Manual of Mental Disorders (DSM-5) (Black and Grant, 2024) to screen for ASD children. Based on the ASD severity classification, it was found that all these children were at Level 1, which means they had relatively mild social communication deficits, for instance, they could engage in simple conversations but had difficulty maintaining in-depth interactions. In addition, restrictive behaviors only occurred when the environment changed, with little impact on their daily lives. Subsequently, the research team used the childhood autism rating scale (CARS) (Rellini et al., 2004) to further assess these children to confirm the diagnosis.

In this study, the 56 included ASD children were randomly divided into an experimental group and a control group, with 28 participants in each group. The random number table method was used in the grouping process to ensure the randomness and balance of the grouping, thereby reducing the impact of inter-group differences on the research results. During the research process, the wishes of the children and their parents were fully respected, and ASD children were only included in the study after obtaining the informed consent form from their parents. At the same time, to protect the privacy and rights of the children, the information of all children participating in the study was strictly confidential, and only code numbers were used in the study. This study was approved by the Scientific Research Ethics Committee of Huaihua University.

### 2.2 Intervention measures

#### 2.2.1 Training of interveners

In this study, the structured martial arts game intervention was implemented by trained interveners. To ensure the standardization and professionalism of the implementation of the structured martial arts game intervention, a systematic training program covering both theoretical and practical aspects was conducted for the interveners. Firstly, theoretical lectures were delivered focusing on the core objectives of the study and the logic of the intervention, covering content such as the design principles of structured martial arts games, the physical and psychological characteristics of children with ASD, and the core framework of the intervention program. Secondly, game process simulation drills were adopted to improve the interveners’ teaching and implementation capabilities. In accordance with the standard process of “10-min warm-up + 40-min game + 10-min relaxation,” game scenarios were set up, and the interveners took turns acting as “intervention implementers” to conduct complete drills of each session’s content, while learning how to explain game rules in language that is easy for children to understand, so as to ensure the smooth connection of the process and its acceptability to children with ASD. In addition, through case-based teaching, the interveners were guided on methods to judge the status of children with ASD and practiced targeted adjustment strategies, ensuring they had the flexible ability to deal with the actual situations of different children. Finally, the interveners’ teaching and implementation capabilities were evaluated through practical assessments, and all interveners eventually passed the assessments.

#### 2.2.2 Design of the intervention

In this study, the experimental group received structured martial arts game interventions, while the control group received traditional rehabilitation training. The rehabilitation effects of structured martial arts games were evaluated by comparing changes in various indicators between the two groups before and after the experiment. Referencing previous systematic reviews (Ji et al., 2023; Teh et al., 2022; Koh, 2024; Wu et al., 2024), the experimental period was set at 24 weeks, with 3 practice sessions per week and each session lasting 60 min. The 60-min schedule included 10 min of warm-up activities, 40 min of structured martial arts game practice, and 10 min of relaxation exercises. During the experiment, training intensity and difficulty were adjusted in a timely manner according to the children’s actual conditions and training progress to ensure the effectiveness and adaptability of the training.

#### 2.2.3 Intervention program for the experimental group

The intervention for the experimental group was structured martial arts game training. A series of structured martial arts games were designed in accordance with the physical and mental characteristics and ability levels of ASD children, adhering to the principles of safety, fun, and pertinence. (1) Safety was the top priority in the design process, with indoor professional sports venues, especially those that were spacious, flat, and obstacle-free, being preferred, and complex and difficult movements like high-risk flips and jumps avoided to prevent injuries and frustration. (2) To enhance the enthusiasm of ASD children for participating in the games, diverse elements such as story backgrounds, role-playing, competition, and cooperation were integrated to increase the games’ fun. (3) The design of the structured martial arts games was highly targeted. Given the significant social communication barriers of children with autism, segments promoting social interaction, such as “martial arts partner practice” and team cooperation tasks, were emphasized to help them learn to pay attention to others, understand intentions, improve communication and collaboration skills, and strengthen team awareness. To address the difficulty ASD children have in maintaining focus (Escobedo et al., 2014), engaging tasks requiring concentration were included, such as the “martial arts memory challenge” where coaches demonstrated a set of moves for children to imitate in sequence, with the number and difficulty of moves increasing gradually to improve their attention and memory. Considering potential sensory integration disorders in ASD children (Krupa-Kotara et al., 2023), the games incorporated multi-sensory stimulation elements, such as setting up obstacles of different heights and slopes in the venue for children to climb over and navigate, stimulating their vestibular and proprioceptive senses, thus helping to improve their sensory integration functions, body coordination, and balance abilities.

Structured martial arts games follow a three-stage implementation process (warm-up, game progression, relaxation) with clear objectives and operational methods, where teachers assume key guiding and supervisory roles. Specific procedures and game examples are detailed in Supplementary Material 1; the core structure is summarized as follows: (1) Warm-up (10 min): Includes jogging, joint mobility exercises, and simple martial arts movements, aiming to activate ASD children’s body joints, raise muscle temperature, prevent sports injuries, and help them gradually enter the game state. (2) Game progression (40 min): Covers basic martial arts movements (kicking, striking, wrestling, grabbing, hitting, stabbing) and integrates fun, situational elements to boost children’s engagement and interest. (3) Relaxation (10 min): Involves slow walking, deep breathing, and simple stretching exercises, designed to relieve children’s physical fatigue, relax their body and mind, and restore a calm state. The implementation process table of structured martial arts games is detailed in Table 1.

**Table 1:** The implementation process table of structured martial arts games.

Stage	Duration	Core content	Objectives
Warm-up	10 min	Jogging, joint movements, and simple martial arts movements	Activate joints, raise muscle temperature, prevent sports injuries, and guide children into the game state
Game progression	40 min	Basic martial arts movements such as kicking, striking, wrestling, grabbing, hitting, and stabbing, integrated with fun and situational elements	Enhance participation and interest, and implement martial arts movement practice
Relaxation	10 min	Slow walking, deep breathing, and simple stretching	Relieve physical fatigue, relax the body and mind, and restore a calm state

#### 2.2.4 Intervention program for the control group

The control group received 24 weeks of traditional rehabilitation training, including conventional rehabilitation methods such as applied behavior analysis (ABA) and speech therapy. (1) APA focuses on improving the social interaction, self-care, and adaptive behavior abilities of children with ASD by enhancing their ability to follow instructions, social response behaviors, and basic life skills. Its core method is the “Discrete Trial Teaching (DTT)” approach, which breaks down complex skills into multiple simple steps and guides children’s learning through a cyclic pattern of “instruction-response-reinforcement.” Each training session lasts 30 min, with 3 sessions per week, interspersed among other rehabilitation methods to ensure the continuity of skill learning. (2) Speech therapy mainly consists of two modules: language comprehension and language expression. Among them, language comprehension training aims to help children establish connections between language and things through real objects, pictures, situational simulation, and other methods; language expression training mainly adopts the “question-guided method” to encourage children to respond with complete sentences, while integrating materials such as picture books and animations to stimulate their interest in language expression. Each training session lasts 30 min, with 3 sessions per week, alternating with APA training to prevent children from feeling fatigued. These methods were implemented by professional rehabilitation therapists following standardized procedures and protocols to ensure that the control group received widely recognized and applied rehabilitation interventions. During the experiment, experimental conditions were strictly controlled to ensure that the two groups were as consistent as possible in all aspects except for the intervention measures.

### 2.3 Variables and tools

In this study, the rater blinding method was adopted for data collection. Firstly, this study selected personnel who had no research interests and possessed professional evaluation capabilities, and conducted special training on data collection for the researchers. Secondly, this study anonymized the participants’ information to prevent the raters from obtaining the participants’ information and ensure the accuracy of the implementation of the blinding method.

#### 2.3.1 Autism treatment evaluation checklist (ATEC)

This study used the autism treatment evaluation checklist (ATEC) (Rimland and Edelson, 1999) to assess the language ability, sensory-perceptual ability, social communication ability, and health behaviors of children with ASD. The scale uses “Always Like This,” “Sometimes Like This,” and “Never Like This” to rate the language ability, sensory-perceptual ability, and social communication ability of children with ASD, with corresponding scores of 0, 1, and 2 points, respectively. For health behaviors of children with ASD, it uses “No Symptom,” “Mild Symptom,” “Moderate Symptom,” and “Severe Symptom,” with corresponding scores of 0, 1, 3, and 4 points, respectively. The total score of the scale is 179 points, where a higher score indicates more severe symptoms in children with ASD (Geier et al., 2013). Reliability and validity tests of the Chinese version of ATEC showed that the Cronbach’s *α* coefficients of the total scale and its subscales ranged from 0.750 to 0.787 (Fang et al., 2019). In addition, the sensitivity of the Chinese version of ATEC’s total scale and subscales ranged from 0.922 to 0.987, the specificity ranged from 0.803 to 0.887, and the area under the curve ranged from 0.924 to 0.972 (Fang et al., 2019). Therefore, the Chinese version of ATEC not only has good internal consistency reliability but also demonstrates high discriminative ability in practical applications, effectively distinguishing individuals with ASD from those without ASD (Fang et al., 2019).

#### 2.3.2 Autism behavior checklist (ABC)

This study used the autism behavior checklist (ABC) (Bravo et al., 2014) to assess sensory ability, social communication ability, motor ability, language ability, and self-care ability in children with ASD. Firstly, it was determined whether the child met the symptoms described in the scale. If “yes,” scoring was conducted sequentially according to the degree of compliance. Each functional score was divided into four levels: “Slightly Consistent,” “Obviously Consistent,” “Basically Consistent,” and “Completely Consistent” with corresponding scores of 1, 2, 3, and 4 points, respectively. If “no,” indicating the child did not exhibit the symptom described, the item was scored 0 points. A total score ≤31 points ruled out ASD, 53 ~ 66 points indicated suspected ASD tendency, and ≥67 points diagnosed ASD (Fernandes and Miilher, 2008). In addition, higher scores indicated more severe ASD symptoms. The scale was completed by parents or caregivers who had lived with the child for at least 6 months (Zheng, 2020). Related studies have confirmed that ABC has a moderate correlation with CARS (Eaves and Milner, 1993), achieving an accuracy rate of over 91% in classifying children with and without ASD (Wadden et al., 1991), demonstrating high reliability and validity.

#### 2.3.3 Gross motor function measure (GMFM)

This study used the gross motor function measure (GMFM) (Harvey, 2017) to assess the postural abilities of children with ASD in five domains: lying and rolling, sitting, crawling and kneeling, standing, and walking and running/jumping, comprising a total of 88 assessment items. GMFM employs a 4-level scoring system (0 points = unable to complete, 1 point = partially completed, 2 points = nearly completed, 3 points = completely completed), where higher scores indicate better motor function. The total scores for each domain are as follows: 51 points for lying and rolling, 60 points for sitting, 42 points for crawling and kneeling, 39 points for standing, and 72 points for walking and running/jumping (Harvey, 2017). Some studies (Brunton and Bartlett, 2011; Russell et al., 2000) have confirmed that GMFM demonstrates high reliability and validity, making it suitable for clinical and research applications.

### 2.4 Mathematical statistics

This study used SPSS 26.0 software to process and analyzes the experimental data. Firstly, mean and standard deviation were used for descriptive statistics of continuous variables, and frequency and percentage were used for descriptive statistics of categorical variables. Secondly, the single-sample K-S test was used, combined with P–P plots and Q-Q plots for normality test of the data. Therefore, the independent samples t-test was adopted to compare the differences in various indicators between the experimental group and the control group before the experiment, so as to test whether the two groups were comparable before the experiment. When performing the independent samples t-test, Levene’s test was used for homogeneity of variance test. If the variance was homogeneous, the results assuming equal variance were used for analysis; if the variance was not homogeneous, the results not assuming equal variance were used. Finally, MANOVA was used to explore the effect of the intervention experiment, where the dependent variables were the main outcome indicators of the pre-test and post-test, and the independent variable was the teaching program (structured martial arts game intervention and traditional martial arts intervention). According to the research purpose, the main effects of the groups and the inter-group effects of the pre-test and post-test were mainly reported. Levene’s test was used for the test of homogeneity of variances; Pillai’s trace was used for the test of model effects; pairwise comparisons were conducted using estimated marginal means (Bonferroni method). In this study, *η*^2^ was used to measure the effect size of MANOVA, where the closer *η*^2^ is to 1, the stronger the influence of the independent variable on the dependent variable; the closer it is to 0, the weaker the influence (Goulet-Pelletier and Cousineau, 2018). Specifically, an *η*^2^ < 0.01 indicates a small effect; 0.01 ≤ *η*^2^ < 0.06 indicates a small effect; 0.06 ≤ *η*^2^ < 0.14 indicates a medium effect; and *η*^2^ ≥ 0.14 indicates a large effect. In addition, due to the large number of outcome indicators and their multiple dimensions, using tables to present data results was not intuitive or aestheticaly pleasing. Therefore, based on GraphPad Prism 8 software, linear trend charts were used to plot the change trends of each indicator after the intervention. The significance level for all the above statistical methods was defined as *α* = 0.05.

## 3 Results

### 3.1 Basic information of participants

All participants took part in the intervention program, with no dropouts or losses to follow-up. After grouping, statistical analysis of the basic information of ASD children in the EG and CG showed no significant differences in age, gender composition ratio, autism severity (*p* > 0.05), indicating comparability between groups. Additionally, there were no statistically significant differences between EG and CG in ATEC, ABC, and GMFM scores (*p* > 0.05). The basic information of the participants is detailed in Table 2.

**Table 2:** Basic information of participants.

Variables	EG (*n* = 28)	CG (*n* = 28)	*t*/*χ^2^*	*p*
Age	6.18 ± 0.61	5.93 ± 0.77	1.349	0.183
Gender (boys: girls)	18: 10	13: 15	1.806	0.215
CARS	37.79 ± 6.14	36.21 ± 3.05	1.214	0.230
ATEC	89.93 ± 5.02	91.29 ± 3.78	−1.143	0.258
Language ability	19.64 ± 2.09	20.61 ± 2.10	−1.722	0.091
Social communication ability	20.79 ± 1.99	21.50 ± 1.48	−1.526	0.133
Sensory-perceptual ability	22.11 ± 1.69	21.46 ± 2.22	1.325	0.227
Health behaviors	27.39 ± 3.43	27.71 ± 2.61	−0.395	0.694
ABC	82.79 ± 3.90	84.39 ± 4.01	−1.520	0.134
Sensory ability	10.39 ± 1.64	10.68 ± 1.39	−0.703	0.485
Social communication ability	19.46 ± 2.08	20.29 ± 1.94	−1.527	0.133
Motor ability	15.96 ± 1.71	16.50 ± 1.73	−1.165	0.249
Language ability	22.29 ± 2.34	21.68 ± 2.14	1.013	0.316
Self-care ability	15.18 ± 0.91	15.25 ± 1.43	−0.223	0.824
GMFM	155.68 ± 4.42	157.11 ± 4.59	−1.186	0.241
Lying and rolling	29.18 ± 2.31	29.82 ± 2.11	−1.087	0.282
Sitting	33.96 ± 1.92	33.11 ± 2.78	1.344	0.185
Crawling and kneeling	23.00 ± 2.47	23.82 ± 1.47	−1.515	0.135
Standing	25.43 ± 1.79	26.18 ± 1.91	−1.516	0.135
Walking and running/jumping	44.32 ± 1.89	44.18 ± 2.33	0.252	0.802

EG, experimental group; CG, control group; CARS, childhood autism rating scale; ATEC, autism treatment evaluation checklist; ABC, autism behavior checklist; GMFM, gross motor function measure.

### 3.2 The changes in various indicators of ATEC after intervention

**Figure 1:**
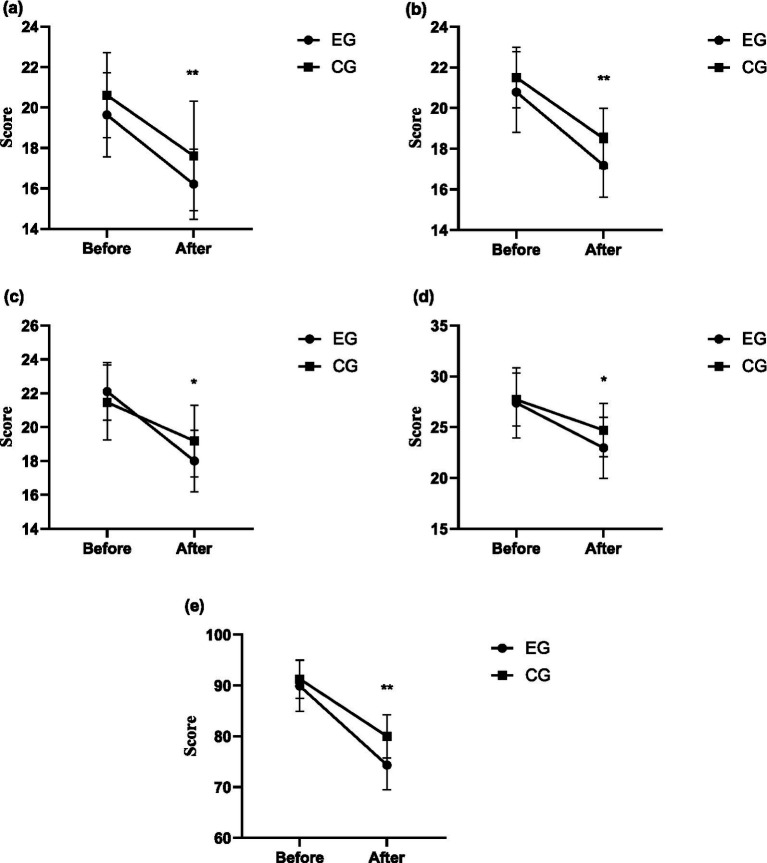
The changing trends of ATEC indicators in the experimental group and the control group after intervention. **(a)** Language ability – ATEC. **(b)** Social communication ability ATEC. **(c)** Sensory-perceptual ability – ATEC. **(d)** Health behaviors – ATEC. **(e)** ATEC. *p < 0.05; **p < 0.01.

For the language ability of children with ASD, the intergroup effect after intervention was significant (*F* = 7.365, *p* = 0.009, *η^2^* = 0.120), and the score of the experimental group (16.21 ± 1.73) was significantly lower than that of the control group (17.61 ± 2.10). For the social ability of children with ASD, the intergroup effect after intervention was significant (*F* = 10.552, *p* = 0.002, *η^2^* = 0.163), and the score of the experimental group (17.18 ± 1.57) was significantly lower than that of the control group (18.50 ± 1.48). For the perceptual ability of children with ASD, the intergroup effect after intervention was significant (*F* = 4.998, *p* = 0.030, *η^2^* = 0.085), and the score of the experimental group (18.00 ± 1.83) was significantly lower than that of the control group (19.18 ± 2.04). For the health behavior of children with ASD, the intergroup effect after intervention was significant (*F* = 5.401, *p* = 0.024, *η^2^* = 0.091), and the score of the experimental group (22.96 ± 3.01) was significantly lower than that of the control group (24.71 ± 2.61). For the total score of ATEC, the intergroup effect after intervention was significant (*F* = 21.220, *p* = 0.000, *η^2^* = 0.282), and the score of the experimental group (74.36 ± 4.85) was significantly lower than that of the control group (80.00 ± 4.30). In summary, the intervention based on structured martial arts games can effectively reduce the obstacles in language, social interaction, perception and health behavior of children with ASD. The intervention based on structured martial arts games achieved a moderate-to-large effect size on the ATEC and all its indicators in children with ASD. The changing trends of ATEC indicators in the experimental group and the control group after intervention are shown in Figure 1.

### 3.3 The changes in various indicators of ABC after intervention

**Figure 2:**
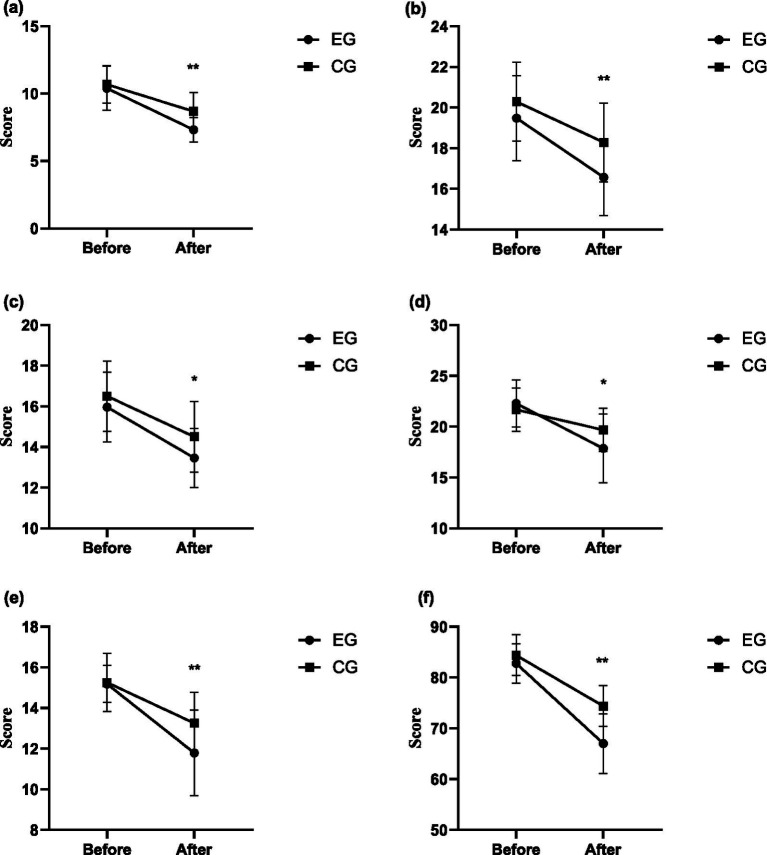
The changing trends of each ABC index between the experimental group and the control group after intervention. **(a)** Sensory ability – ABC. **(b)** Social communication ability – ABC. **(c)** Motor ability – ABC. **(d)** Language ability – ABC. **(e)** Self-care ability – ABC. **(f)** ABC. *p < 0.05; **p < 0.01.

For sensory ability, the intergroup effect after intervention was significant (*F* = 18.762, *p* = 0.000, *η*^2^ = 0.258), and the score of the experimental group (7.32 ± 0.91) was significantly lower than that of the control group (8.68 ± 1.39). For social ability, the intergroup effect after intervention was significant (*F* = 11.188, *p* = 0.002, *η*^2^ = 0.172), and the score of the experimental group (16.57 ± 1.89) was significantly lower than that of the control group (18.31 ± 2.01). For motor ability, the intergroup effect after intervention was significant (*F* = 5.878, *p* = 0.019, *η*^2^ = 0.098), and the score of the experimental group (13.46 ± 1.45) was significantly lower than that of the control group (14.52 ± 1.88). For language ability, the intergroup effect after intervention was significant (*F* = 5.785, *p* = 0.020, *η*^2^ = 0.087), and the score of the experimental group (17.86 ± 3.89) was significantly lower than that of the control group (19.62 ± 2.22). For self-care ability, the intergroup effect after intervention was significant (*F* = 9.212, *p* = 0.004, *η*^2^ = 0.146), and the score of the experimental group (11.79 ± 2.11) was significantly lower than that of the control group (13.30 ± 1.48). For ABC, the intergroup effect after intervention was significant (*F* = 30.456, *p* = 0.000, *η*^2^ = 0.361), and the score of the experimental group (67.00 ± 5.84) was significantly lower than that of the control group (74.31 ± 4.11). In summary, the intervention based on structured martial arts games can effectively reduce the behavioral disorders of sensory, social, motor, language, self-care, etc. in children with ASD. The intervention based on structured martial arts games achieved a moderate-to-large effect size on the ABC and all its indicators in children with ASD. The changing trends of each ABC index between the experimental group and the control group after intervention are shown in Figure 2.

### 3.4 The changes in various indicators of GMFM after intervention

**Figure 3:**
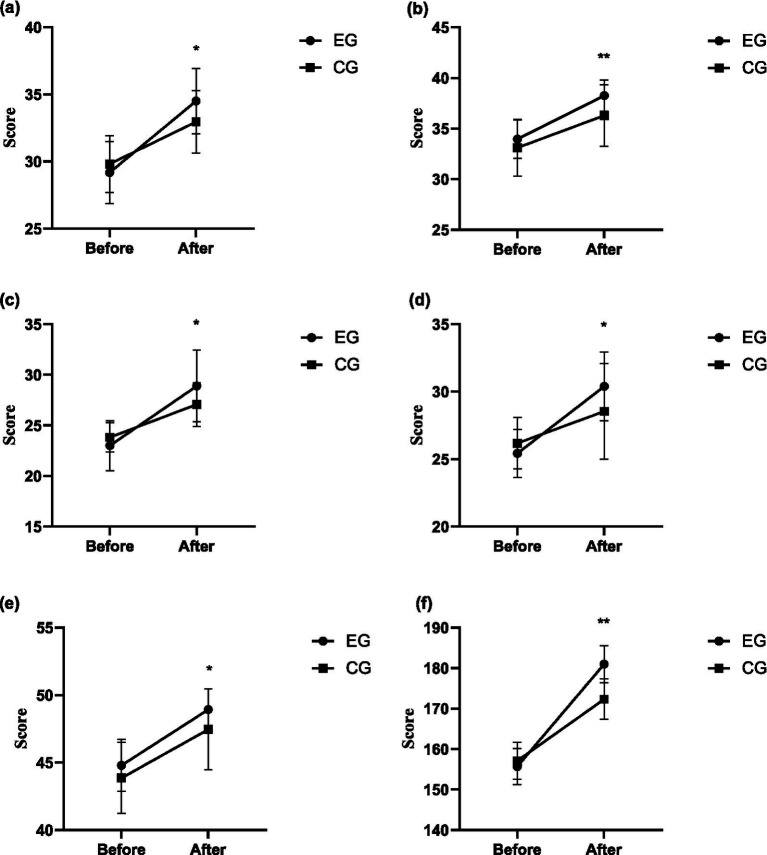
The changing trends of GMFM indicators in the experimental group and the control group after intervention. **(a)** Lying and rolling – GMFM. **(b)** Sitting – GMFM. **(c)** Crawling and kneeling – GMFM. **(d)** Standing – GMFM. **(e)** Walking and running/jumping – GMFM. **(f)** GMFM. *p < 0.05; **p < 0.01.

For lying position and turning over, the intergroup effect after intervention was significant (*F* = 6.065, *p* = 0.017, *η^2^* = 0.101). The score of the experimental group (34.50 ± 2.43) was significantly higher than that of the control group (32.96 ± 2.34). For sitting position, the intergroup effect after intervention was significant (*F* = 9.203, *p* = 0.004, *η^2^* = 0.146). The score of the experimental group (38.25 ± 1.56) was significantly higher than that of the control group (36.29 ± 3.05). For crawling and kneeling, the intergroup effect after intervention was significant (*F* = 5.423, *p* = 0.024, *η^2^* = 0.091). The score of the experimental group (28.89 ± 3.53) was significantly higher than that of the control group (27.07 ± 3.04). For standing position, the intergroup effect after intervention was significant (*F* = 5.057, *p* = 0.029, *η^2^* = 0.086). The score of the experimental group (30.39 ± 2.56) was significantly higher than that of the control group (28.54 ± 3.54). For walking and running/jumping, the intergroup effect after intervention was significant (*F* = 5.353, *p* = 0.025, *η^2^* = 0.090). The score of the experimental group (48.93 ± 1.54) was significantly higher than that of the control group (47.46 ± 2.98). For GMFM, the intergroup effect after intervention was significant (*F* = 45.576, *p* = 0.000, *η^2^* = 0.458). The score of the experimental group (180.96 ± 4.59) was significantly higher than that of the control group (172.32 ± 6.45). In conclusion, the intervention based on structured martial arts games can effectively improve students’ motor functions such as lying position and turning over, sitting position, crawling and kneeling, standing position, walking, running and jumping. The intervention based on structured martial arts games achieved a moderate-to-large effect size on the GMFM and all its indicators in children with ASD. The changing trends of GMFM indicators in the experimental group and the control group after intervention are shown in Figure 3.

## 4 Discussion

### 4.1 Analysis of the main findings

This study deeply explored the rehabilitation effects of structured martial arts games on children with ASD through a randomized controlled trial. It was found that the intervention based on structured martial arts games can effectively improve the behavioral and functional disorders of children with ASD. This study provides a new and effective intervention method for the rehabilitation training of children with ASD. The results of this study are similar to previous research on exercise interventions for social skills (Jia et al., 2023; Koh, 2024) and fundamental motor skills (Ji et al., 2023) in children with ASD, all demonstrating positive intervention effects. A meta-analysis by Wu et al. (2024) found that martial arts skill training exerts a positive intervention effect on social deficits and repetitive stereotyped behaviors in children with ASD. Additionally, Jia et al. (2023) categorized physical exercise types into individual exercise groups and group exercise groups. Through a quantitative meta-analysis, they revealed that the difference between the group exercise group and the control group was statistically significant, while there was no statistically significant difference between the individual exercise group and the control group. This further supports the complex interaction factors in the structured martial arts games of this study. The improvement effect of structured martial arts games on behavioral and motor disorders in children with ASD is not the result of single-dimensional intervention, but through the multi-system integration of neurodevelopment, behavior shaping, and functional improvement, achieving synergistic improvement of multiple indicators such as behavioral and motor disorders in children with ASD.

Firstly, at the neurodevelopmental level, the intervention based on structured martial arts games can promote the remodeling of multi-sensory-motor loops in children with ASD, and enhances the secretion of neurotransmitters such as dopamine and endorphin (da Silva Duarte et al., 2023; Wojciechowski et al., 2021). This provides a neurophysiological basis for improving behavioral and functional disorders in children with ASD. Martial arts movements integrate tactile, proprioceptive, vestibular, and visual–spatial perception to form multi-channel neural stimulation (Wang et al., 2025). During the learning and practice of various movements, children need to simultaneously integrate visual target positioning, proprioceptive limb control, and tactile feedback. This integrated input can repair the damaged sensory integration neural pathways in children with ASD (Preis and McKenna, 2014), reducing behavioral and motor disorders caused by abnormal sensory processing. The auditory reception-motor execution process of martial arts-related action commands can also activate the neural connection between the language area of the left brain hemisphere and the motor cortex (Pulvermüller et al., 2005). Eye contact and limb coordination in partner training promote the development of the prefrontal social cognitive network (Sudo et al., 2020; Mu et al., 2018). This neural coordination across brain regions may contribute to the simultaneous improvement of social, motor, and communication skills. In addition, regular martial arts exercise can effectively promote the secretion of dopamine and endorphin, alleviate anxiety in children with ASD, and reduce repetitive stereotypic behaviors caused by serotonin imbalance (Naves-Bittencourt et al., 2015; Tabeshian et al., 2022).

Secondly, the intervention that draws on structured martial arts games is characterized by well-defined structural rules and a wide variety of game forms. Through the dual reinforcement of rules and games, it can effectively reshape the behavioral and motor disorders of children with ASD. This study transformed martial arts movements into a series of game-based tasks, enhancing the motivation of children with ASD to participate through mechanisms such as point-based rewards and role-playing. For example, in the “Monster Target Striking” game, children with ASD will actively repeat standard kicking movements to obtain rewards. This reinforcement process not only improves the lower limb motor ability of children with ASD but also reduces inattention through goal-oriented behaviors. In addition, game-based tasks in partner training require children to take turns performing movements and providing feedback to peers. This integrated “social-motor” scenario directly simulates daily life interactions. For instance, children need to suppress the urge to cut in line while waiting for peers to complete movements; high-fives during team training reinforce the neural memory of positive social behaviors, achieving a social progression from “passive cooperation” to “active initiation.”

Finally, in terms of functional improvement, the intervention based on structured martial arts games achieves hierarchical integration from basic motor skills to social adaptation, effectively enhancing the behavioral and motor functions of children with ASD. This study designed training involving various basic motor controls. For example, the “horse stance” can directly improve sitting stability; movements such as “bear crawling” can directly enhance limb coordination and improve crawling and kneeling skills in children with ASD. The study also designed multiple complex motor integration tasks. For instance, combined movements like “side lunge movement + punch” can promote standing balance and gait coordination in children with ASD; sensory integration games such as blindfolded target kicking enhance spatial orientation through the linkage of tactile and proprioceptive senses.

In conclusion, the reason why structured martial arts games can effectively improve behavioral and motor disorders in children with ASD lies in the construction of a three-dimensional integrated intervention system of “neurodevelopment-behavior shaping-functional improvement”: activating neural plasticity through multi-sensory movements, inhibiting abnormal behaviors with structured rules, and promoting functional migration with gamified social interactions, ultimately achieving synergistic improvement of multiple indicators such as ATEC, ABC, and GMFM. This integrated model not only provides a new path for ASD rehabilitation, but also reveals the interaction mechanism of “physical activity-cognitive development-social adaptation,” offering a theoretical reference combining scientific and practical value for the field of special children’s intervention.

### 4.2 Implications and applications of the research findings

The results of this study show that structured martial arts games have a significant effect on the rehabilitation of children with ASD, which provides many enlightenments for the rehabilitation training of children with ASD and has important guiding significance in practical applications.

The research findings suggest that structured martial arts games, as an innovative rehabilitation intervention method, have unique advantages and are worthy of wide promotion and application in the field of ASD children’s rehabilitation. Their fun and attractiveness can effectively stimulate the participation enthusiasm of children with ASD, making them more proactive in investing in rehabilitation training. By participating in martial arts games, children with ASD can receive comprehensive exercise and development in terms of behavior and motor functions. Relevant departments and institutions should strengthen the publicity and promotion of structured martial arts games, so that more children with ASD and their families can understand and benefit from this rehabilitation method. Demonstration courses and training activities of structured martial arts games can be organized, and professional martial arts coaches and rehabilitation experts can be invited to provide guidance to improve the understanding and application ability of rehabilitation workers regarding structured martial arts games. Special education schools, ASD rehabilitation institutions, etc., are encouraged to incorporate structured martial arts games into the daily rehabilitation training curriculum system to provide more opportunities for children with ASD to participate in martial arts games.

In addition, schools and communities should also play an active role in the rehabilitation process of children with ASD. Schools can incorporate structured martial arts games into physical education courses or extracurricular activities, providing more opportunities for children with ASD to participate in group activities and promoting their integration with typical children. Activities such as martial arts interest groups and martial arts clubs can be organized to help children with ASD improve their social skills and self-confidence through interaction with peers. Communities can host various martial arts-related activities, such as martial arts performances and lectures, and invite children with ASD and their families to participate, creating an inclusive and supportive community environment. Communities can also establish volunteer service teams to provide assistance and support to families of children with ASD, helping them carry out structured martial arts game training.

### 4.3 Limitations of this study

Although this study provides a program of structured martial arts games that can effectively improve the behavioral and functional disorders of children with ASD, there are also certain limitations in sample representativeness, intervention design, and evaluation methods. First, the sample size of this study is relatively small, and all participants are from a special education school in Chengdu, which may not be able to represent children with ASD of different ages, regions, cultural backgrounds, or socioeconomic development levels, thus limiting the universality of the research results. Second, the intervention period of this study is 24 weeks, which covers short-term effects, but lacks long-term follow-up data (such as follow-up for more than 6 months after the intervention ends), making it impossible to confirm whether the effects of structured martial arts games are sustainable. Finally, this study mainly relies on caregiver reports and observer evaluations, lacking the subjective feedback of children with ASD, which may lead to reporting bias. In addition, GMFM focuses on gross motor skills but does not involve more subdivided dimensions such as fine motor skills and postural control, so the comprehensiveness of the evaluation system needs to be improved. Moreover, neuroscientific indicators (such as electroencephalography and functional magnetic resonance imaging) have not been used, making it impossible to deeply analyze the mechanism of action of structured martial arts games on the neurodevelopment of children with ASD.

Based on this, this study suggests carrying out multi-center and large-sample studies covering children with ASD of different ages, regions, and comorbidity types to verify the universality of the intervention effect; it is recommended to increase long-term follow-up after the intervention to observe the sustainability of improvements in behavior and motor function and clarify the long-term benefits of structured martial arts games. In addition, this study suggests introducing fine motor assessment scales and social–emotional assessment tools to build a more comprehensive intervention effect evaluation system; it is recommended to combine neuroimaging techniques (such as fMRI) and biomarker detection (such as dopamine and endorphin levels) to explore the impact of martial arts games on brain plasticity and neurotransmitter secretion in children with ASD, so as to improve the interpretability of the research results.

## 5 Conclusion

This study has confirmed through a randomized controlled trial that structured martial arts game intervention can effectively improve the behavioral and motor function disorders of children with ASD. This suggests that structured martial arts games, as an innovative intervention method, not only have the characteristics of fun and structure, but also can significantly stimulate the participation enthusiasm of children with ASD, providing a new feasible path for ASD rehabilitation. It is recommended that relevant departments and institutions strengthen promotion efforts, incorporate them into the regular curricula of special education schools and rehabilitation institutions, and create a supportive environment through community activities (such as martial arts performances and volunteer services) to promote the social integration of children with ASD. In addition, it is recommended to add long-term follow-up after the intervention to observe the sustainability of the improvement effects on behavioral and motor functions, and to clarify the long-term benefits of structured martial arts games. Moreover, it is recommended that future research should carry out multi-center large-sample trials covering ASD populations of different ages and regions, combine neuroimaging techniques and biomarker detection to deeply explore the neural mechanisms of intervention, and optimize personalized intervention programs to enhance the universality of research results and the precision of intervention.

## Data Availability

The raw data supporting the conclusions of this article will be made available by the authors, without undue reservation.
